# The Use of Calcium Phosphates in Cosmetics, State of the Art and Future Perspectives

**DOI:** 10.3390/ma14216398

**Published:** 2021-10-25

**Authors:** Francesca Carella, Lorenzo Degli Esposti, Alessio Adamiano, Michele Iafisco

**Affiliations:** Institute of Science and Technology for Ceramics (ISTEC), National Research Council (CNR), Via Granarolo 64, 48018 Faenza, Italy; francesca.carella@istec.cnr.it (F.C.); lorenzo.degliesposti@istec.cnr.it (L.D.E.); alessio.adamiano@istec.cnr.it (A.A.)

**Keywords:** hydroxyapatite, oral care, skin care, sunscreens, deodorants, hair care

## Abstract

Calcium phosphates (CaPs) belong to a class of biomimetic materials widely employed for medical applications thanks to their excellent properties, such as biodegradability, biocompatibility and osteoinductivity. The recent trend in the cosmetics field of substituting potentially hazardous materials with natural, safe, and sustainable ingredients for the health of consumers and for the environment, as well as the progress in the materials science of academics and chemical industries, has opened new perspectives in the use of CaPs in this field. While several reviews have been focused on the applications of CaP-based materials in medicine, this is the first attempt to catalogue the properties and use of CaPs in cosmetics. In this review a brief introduction on the chemical and physical characteristics of the main CaP phases is given, followed by an up-to-date report of their use in cosmetics through a large literature survey of research papers and patents. The application of CaPs as agents in oral care, skin care, hair care, and odor control has been selected and extensively discussed, highlighting the correlation between the chemical, physical and toxicological properties of the materials with their final applications. Finally, perspectives on the main challenges that should be addressed by the scientific community and cosmetics companies to widen the application of CaPs in cosmetics are given.

## 1. Introduction

Cosmetics, as defined by the U.S. Food and Drug Administration (FDA), are substances for application to the human body aimed at cleansing, beautifying, promoting attractiveness or altering the appearance without affecting the body physiology or functions [1]. In particular, it refers to every substance placed in contact with an external part of the human body or with the teeth and the mucous membranes of the oral cavity with the aim of cleaning, protecting, perfuming or changing their appearance [2,3,4].

The first use of the term “cosmetics” dates to the Greek term “kósmesis” that means “to order” or “to adorn”. In ancient times, cosmetics were handled by males and females of all ages in everyday life for aesthetic reasons, in religious rituals, or for medical purposes. Cosmetic ingredients were obtained from several vegetal, animal, or mineral sources. Some of the animal and vegetal ingredients were, for instance, egg whites, ground animal bones mixed with oils (either almond or poppy), lemon juice, roses, camphor, wax, oyster shells, red dye from cochineal, or the slime of snails [5,6]. Mineral cosmetics were instead hematite (Fe_2_O_3_), galena (PbS), cerussite (PbCO_3_), laurionite (PbOHCl), malachite (Cu_2_(CO_3_)(OH)_2_), mercury oxide (HgO), zinc oxide (ZnO), and silver or gold foils [6,7]. It is interesting to note that many of these ingredients of natural origin are still commonly used in modern cosmetics.

Due to the rapid development of the petrochemical industry over the last two centuries, nowadays synthetic cosmetic materials are mass-produced and diffused worldwide [8]. During the XX century, toxic heavy metals were found in cosmetics as contaminations or impurities. Various research works report a direct relationship between chronic exposure to heavy metals in cosmetics and health problems such as skin sensitivities, allergic reactions, contact dermatitis, hair loss, respiratory disorders, cardiovascular diseases, gastrointestinal disorders, fertility problems, cancer, and even death [9,10]. The European Regulation no.1223/2009/EC permits the presence of traces of heavy metals in the finished products if it is technically inevitable. Such presence is tolerated only if the safety of the product is demonstrated and if good manufacturing practices are employed. However, in the regulation there are no precise limits of the quantity of metals tolerated in a cosmetic product, even if as a rule of thumb their concentration must be kept as low as possible. Indeed, every government is demanded to enforce limits on impurities content in cosmetic products on sale. An example is reported by the German Federal Government, which has determined appropriate limits for metal contained as impurities in cosmetics, such as 5 µg/g for As and Cd, 1 µg/g for Hg, 20 µg/g for Pb and 10 µg/g for Sb [11,12]. Another example is represented by the limits for elements present as impurities in cosmetic products imposed by the Government of Canada, which are 3 µg/g for As, Cd and Hg, 10 µg/g for Pb, and 5 µg/g for Sb [11,13].

The safety of cosmetics means not only to avoid as much as possible the presence of heavy metals, but also to ensure that the product has an adequate microbiological purity and stability [14]. For this purpose, producers usually use preservatives such as parabens, which are biodegradable and do not change the consistency or color of products. According to different regulatory agencies such as the FDA and the European Chemicals Agency (ECHA), parabens are considered safe, but several works report that a continuous use of cosmetics containing this class of compounds can be harmful for the human body [2,15,16,17,18].

Other categories of compounds used in cosmetics for several purposes, such as UV filters for their photoprotective action or microplastics for their texture-extending and feel-modifying abilities, raise concerns both from the consumer health and environmental perspectives. For instance, in the marine environment, these chemicals can damage fragile and precious ecosystems such as coral reef, causing a loss of biodiversity, and can bioaccumulate in the consumed fish species, again endangering human health.

To address these health and environmental concerns, it is therefore necessary to produce and use innovative, natural, and safe ingredients that have high biocompatibility and biodegradability and are non-toxic. A class of materials that has all these requisites is represented by calcium phosphates (CaPs), whose application in cosmetics is raising interest and will be discussed and analyzed in detail in this review.

## 2. Calcium Phosphates

CaPs are a family of materials and minerals that constitute the inorganic component of hard tissues in vertebrates (e.g., bones and teeth) and are also present in milk and blood as the principal mineral of calcium [19,20]. Nowadays, the main application of CaPs is in medicine, where they are used as biomaterials in orthopedics for regenerating or replacing bone tissue thanks to their excellent biocompatibility, bioactivity and bioresorbability [21]. Synthetic CaPs can be recognized by the body as a sort of endogenous material due to their chemical and structural similarity to the mineral phase of bone, and this is the reason for their high biocompatibility. Indeed, in comparison to other materials used in orthopedics, CaPs have a higher osteoconductivity and osteoinductivity, do not induce immune responses or rejections, and can stimulate bone self-healing. In the last 50 years CaPs were used as: (i) bone replacement as three-dimensional massive bioceramics or scaffolds, (ii) as injectable self-hardening cements to fill bone defects for setting exogenous implants, (iii) as filling material in soft bio-composites and hybrid biomaterials, or (iv) as coating for metallic and polymeric protheses [22,23,24,25,26]. In the case of massive CaP bioceramics, a limitation for their wider use in clinical application is related to their poor mechanical properties. Pure CaPs are characterized by being fragile, as they have both low impact resistance and low tensile stress (6 to 10 MPa), making them unsuitable for replacing bone (which has a tensile strength of 50 to 150 MPa in the case of cortical bone) [21]. For these reasons, they can be used in the form of powder or cements combined with several elements, such as Fe, Ag, Cu, Mg, Mn, Sr or Zn, or are reinforced with polymers to form bio-hybrid composites [27,28,29,30,31,32]. One of the most successful applications of CaPs is as coatings of metallic or polymeric implants that have poor osteoconductivity to improve their integration with the bone [33,34,35,36,37,38]. The CaP coating can initiate a bioactive fixation of the prothesis after surgery and increase the long-term activity of the implant, and at the same time limit the fibrous tissue encapsulation around it. In addition, CaPs in the form of nanoparticles (NPs) were recently proposed as innovative materials in nanomedicine, to treat diseases not related to bone (i.e., cardiovascular diseases, cancer, etc.), harnessing their superior biocompatibility for drug delivery applications [39,40,41,42]. One of the main aims of the drug delivery systems is to convey a poorly bioavailable drug to the target tissue using the capability of a nanomaterial to cross biological barriers and to avoid its early clearance [43]. CaP NPs are interesting vectors for drug delivery since they can load a high variety of bioactive molecules, thus protecting the therapeutic agent from degradation in the biological environment.

**Figure 1:**
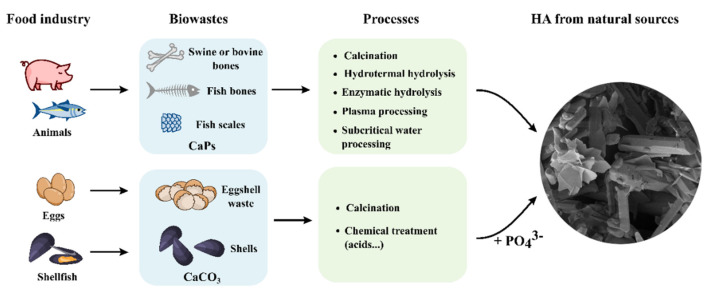
Scheme of the preparation of calcium phosphates from biogenic sources.

As mentioned before, CaPs are widely present in nature and in particular as minerals in vertebrate bones, mammalian teeth, and fish scales. Thus, CaPs cannot be only synthesized by chemical reactions, but they can be also prepared in several ways from biogenic sources such as eggshells, bones, and seashells (Figure 1). The conversion of these food industry by-products to compounds with high added value, applying the principles of circular economy, is nowadays a significant topic for social, environmental, and economic reasons. CaPs from bones are commonly obtained by removing all the organic components by thermal treatment [44], while CaPs from eggshells or seashells that are made of calcium carbonate are usually prepared with a two-step process based on thermal treatment to convert CaCO_3_ in CaO and subsequent precipitation with a phosphorus source [45].

**Figure 2:**
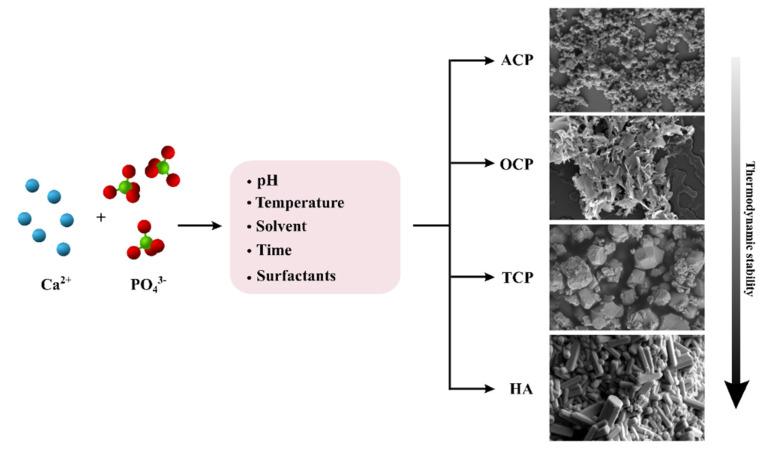
Principal synthesis parameters that influence the characteristics of calcium phosphates as well as the formation of different crystal phases (ACP: amorphous calcium phosphate, OCP: octacalcium phosphate, TCP: tricalcium phosphate, HA: hydroxyapatite).

Irrespective of the application, all the functions of CaPs are tightly related to their physicochemical characteristics, such as particle size, morphology, crystallinity, porosity, density, composition, Ca/P atomic ratio, or pH stability range, and all these characteristics can be tailored by modifying the CaP synthesis (Figure 2) [26,46,47]. In addition, different CaP crystal phases can be prepared, which in turn have different morphologies, chemical compositions, or structures [26,46,47]. In this section, the main proprieties of the four more relevant CaP phases for cosmetic applications will be described.

### 2.1. Amorphous Calcium Phosphate

Amorphous calcium phosphate (ACP) is a non-crystalline CaP phase and represents the mineral precursor for bone and tooth formation in vertebrates [48,49]. As the other CaP phases, ACP is bioactive, osteoconductive and has found application as bone repair material in cements, for coatings of metallic or polymeric bone implants and as a drug delivery platform [50,51]. In addition, being a non-crystalline phase, ACP is more soluble than the crystalline CaPs and it can release a high amount of calcium and phosphate ions in a short time span. This property has been harnessed in the dental field, leading to the development of ion-releasing toothpastes containing ACP that trigger enamel and dentin remineralization. Indeed, the high concentration of ions in the oral environment generated by ACP-containing products induces the formation of a new mineral phase onto the dental tissue, restoring the mineral loss caused by caries [52]. Commonly, ACP is obtained by a wet precipitation in an aqueous environment, even though precipitations in ethanol or by sol-gel processes were also reported [50]. In order to produce an amorphous product, it is necessary to use high supersaturation conditions, additives, and fast precipitation times. ACP does not have a precise stoichiometry, and on the basis of the precipitation conditions its Ca/P molar ratio ranges from 1.18 to 2.50 [53]. ACP is highly unstable, and it easily transforms into crystalline CaPs. This is due to the high structural similarity in the short-range order of ACP with octacalcium phosphate (OCP) and hydroxyapatite (HA), and in the presence of water or moisture the amorphous structure rearranges spontaneously to form a crystalline lattice. The crystallization process is affected by many factors, such as pH, temperature, humidity, and the presence of ions/additives [50,53]. As the superior ion-releasing properties of ACP are lost with the spontaneous crystallization, many studies were conducted to stabilize ACP in the long term to enhance its shelf-life. The most common stabilizers of ACP are ions such as Mg^2+^, CO_3_^2−^ and P_2_O_7_^4−^, as they hinder crystalline lattice formation [54,55]. Otherwise, other common stabilizers are organic molecules that attach to the ACP surface and inhibit the dissolution–reprecipitation mechanisms of crystallization. Among them, the most successful stabilizers are casein phosphopeptide, which is a milk protein, or citrate, which is a relatively abundant organic molecule of bone [21,24,50,53,56,57,58,59,60].

### 2.2. Hydroxyapatite

Hydroxyapatite (HA) has the formula of Ca_10_(PO_4_)_6_(OH)_2_ with a Ca/P ratio of 1.67, is the most thermodynamically stable CaP phase in physiological conditions and is the mineral phase of vertebrate bones, mammalian teeth, and fish scales. HA is characterized by several features, such as superior bioactivity, osteoconductivity, non-toxicity, non-immunogenicity, and biocompatibility in comparison to other CaPs [61]. These properties are enhanced when HA is synthesized to have the same crystallinity, chemical composition, ion doping, size, and morphology of the biogenic minerals, which in this case is defined as biomimetic HA [61]. Usually, biological and biomimetic HAs are non-stoichiometric, contain foreign doping ions in their hexagonal structure such as Na^+^, K^+^, Mg^2+^, Sr^2+^, Fe^2+^/^3+^, Zn^2+^, CO_3_^2−^, Cl^−^, or F^−^, as the HA structure is flexible, and can accommodate many ions by the substitution of Ca^2+^, PO_4_^3−^, or OH^−^ ions [62]. Indeed, HA has been doped with monovalent, divalent, and trivalent cations by substitution of calcium ions, and with divalent or trivalent anions by substitution of phosphate ions; in addition, hydroxyl ions can also be substituted with monovalent or divalent anions [62]. Furthermore, some dopant ions can also be introduced in interstitial position of the crystal lattice. These ionic substitutions can greatly alter HA properties, e.g., increase HA solubility, bioactivity, stability, or impart additional capabilities such as magnetic sensitivity or luminescence [21,56,57,63,64]. A peculiar feature of HA is its pH-dependent water solubility, as it is stable in alkaline solutions, poorly soluble at neutrality and soluble at acidic pH. Therefore, this pH sensitivity can be harnessed for pH-triggered drug delivery applications, where a drug associated to HA is released only when the material encounters an acidic environment, as in the cases of inflammatory regions or in endosomes and lysosomes after cellular intake [42]. Finally, it has also been demonstrated that HA nanocrystal size and morphology can significantly affect its biocompatibility, bioactivity, and cell and tissue penetration capability. HA is a tailorable material, and there is a wide array of methods for its preparation to obtain different products, which were exhaustively reported in the work of Sadat-Shojai et al. [65].

### 2.3. Octacalcium Phosphate

Octacalcium phosphate (OCP) is a CaP with formula Ca_8_H_2_(PO_4_)·6.5H_2_O, and is thought to be a precursor of biogenic HA in hard tissues of vertebrates [66]. Indeed, OCP triclinic structure is very similar to the hexagonal structure of HA, as it is composed of “apatitic layers” that have the same atomic arrangement of HA intercalated by “hydrated layers” that contain water molecules [21,56,57,67,68,69]. As a consequence of this, OCP has good osteoconductivity and it can convert into HA through a dissolution–reprecipitation mechanism or a topotaxial conversion mechanism [67], and for these reasons it has found successful application in bone-related biomaterials [21,56,57,67,68,69]. It has also been proposed that the HA of bones is formed by conversion of an OCP precursor [67]. Synthetic OCP can be obtained by wet precipitation at neutral or mildly acidic pH or by the hydrolysis of α-TCP or dicalcium phosphate [67]. Interestingly, the Ca/P molar ratio of OCP (1.33) is variable and can change according to the quantity of calcium present in the structure. Indeed, its structure could be both Ca-deficient (Ca/P 1.26), or it can include an excess of calcium (Ca/P up to 1.48) [26].

### 2.4. Tricalcium Phosphate

Tricalcium phosphate (TCP) is characterized by the chemical formula Ca_3_(PO_4_)_2_ and a Ca/P ratio of 1.50 [26]. TCP exists in two allotropic forms that have the same chemical composition but different structure, density, and solubility: α-TCP and β-TCP. The latter allotropic form is the more thermodynamically stable, but usually is further stabilized by including magnesium ions into calcium ion vacancy. β-TCP has a lower interfacial energy than HA, and for this reason, in aqueous ionic solutions, it can induce the precipitation of an apatitic layer. Furthermore, it is osteoconductive and osteoinductive [70]. α-TCP has similar properties, but it is more soluble and reactive than β-TCP [21,56,57,63,71,72]. Usually, TCPs are obtained by the high-temperature solid-state reaction of solid Ca and P precursors (e.g., calcium carbonate and ammonium hydrogen phosphate), or by the thermal transformation of CaP precursors with a Ca/P molar ratio equal to 1.50 that can be calcium-deficient hydroxyapatite or ACP. Depending on the reaction temperature, a different allotropic form can be produced. Indeed, to obtain β-TCP, the CaP precursors are calcined at 700–800 °C, while to achieve α-TCP the process temperature increases up to 1200 °C. In addition, α-TCP could also be prepared by thermal transformation from crystalline β-TCP, and this is the most direct and simplest approach to produce α-TCP [71,73,74,75]. The main medical application of TCP, in particular α-TCP, is as self-setting CaP cements. Cements are formed when TCP or a blend of CaP powders are mixed with an aqueous solution, obtaining a viscous paste that can be injected in the bone defect and progressively hardens in situ, restoring mechanical resistance [76]. The hardening is due to the hydrolysis of TCP that recrystallizes into HA, as the newly formed crystallites interlock with one another during growth, forming a hard conglomerate [77].

## 3. Applications of Calcium Phosphates in Cosmetics

In the last few years, CaPs have been used for different applications besides the medical field, such as controlled-release fertilizer and bio-stimulant in agriculture, catalyst, anticorrosive material for paintings, flame retardant agent, etc. [78,79,80,81]. Indeed, the excellent properties of CaPs that made them a versatile material in medicine can also be useful in the cosmetics field. First and foremost, the excellent biosafety of CaPs allows one to substitute cosmetic ingredients raising health concerns with safer alternatives. In addition, the compositional flexibility and high variability of CaP properties permits one to design materials that can fulfil different functions requested by the cosmetics industry.

**Figure 3:**
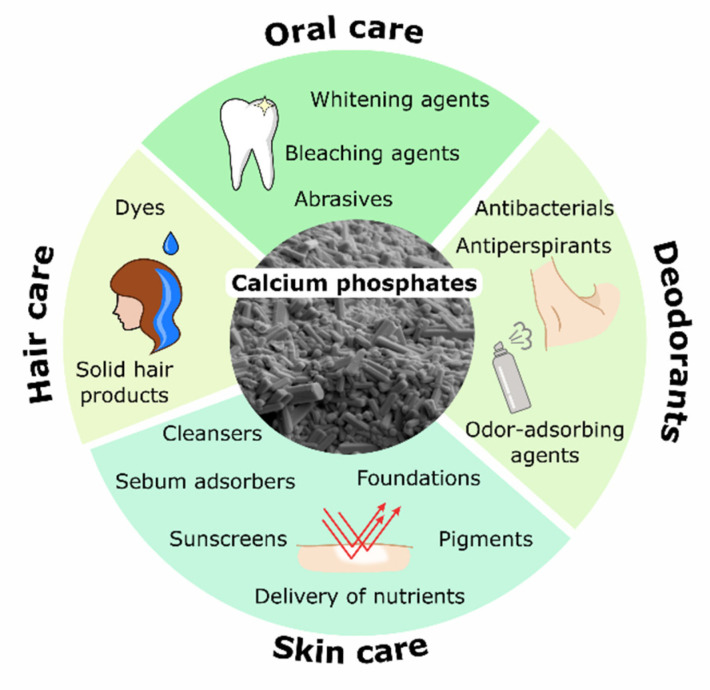
Current and potential application of calcium phosphates in the cosmetics field.

The purpose of this review is to provide an overview of the state of the art of the use of CaPs as cosmetic ingredients and to highlight future challenges and trends in this topic. In the next sections, we will report the importance of CaP phases in cosmetics as innovative and safe materials and the research works in this field. The main applications of CaPs as a cosmetic ingredient reported in the scientific literature that we have found are in oral care, skin care, hair care, and deodorants (Figure 3).

**Figure 4:**
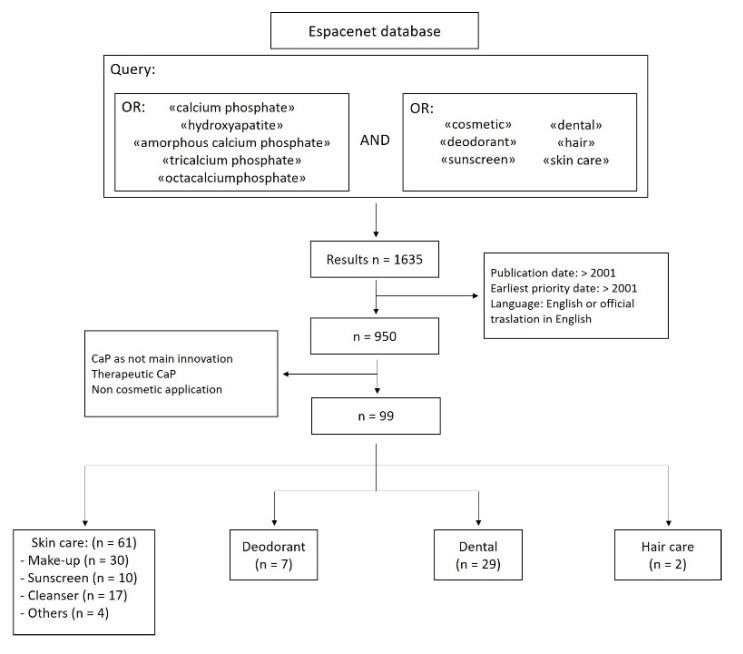
Flowchart of the patent search and refinement.

As a matter of fact, there is a rich body of literature on patents about CaPs for cosmetic applications and, for this reason, the most significant patents in the field are also reported and the general trends are discussed. In particular, we performed a patent search with the open database Espacenet of the European Patent Office [82], and subsequently we refined the result according to the flowchart reported in Figure 4. Briefly, we searched all the patents that present the combination of terms between the most relevant CaP phases (HA, ACP, TCP, OCP, and the generic term “calcium phosphate”) and the broadest terms for the cosmetics industry (cosmetics, oral, hair, sunscreen, skin care, deodorant), obtaining 1635 hits from the query. Afterward, we refined the results by eliminating the patents that were not filled in English and for which there is not an official English translation, or were already expired, thinning down the list to 950 patents. Finally, we have manually sorted the patents excluding the ones that were not appropriated, that is to say the ones where CaP was not a key element to the innovation but just a generic ingredient, and the ones that had only medical applications. In this way, the final number of relevant patents was 99. The patents were divided in the same categories as the research articles reported above, obtaining 61 patents for skin care materials, 7 for deodorant materials, 29 for dental whitening material, and 2 for hair care materials.

### 3.1. Oral Care

Oral care is a sector that lies in between health and cosmetics. CaPs are widely known for their efficacy as remineralizing agents, desensitizing agents, and anti-caries materials [83,84]. However, these medical applications are not considered in this work.

The tooth is composed of an outer mineral layer, the enamel, that covers an inner layer of dentin. Both enamel and dentin are mineralized tissues and are mainly composed of HA and collagen. In enamel, HA is more crystalline and the organic matter is less than 1 wt.%, while in dentin HA is less crystalline and organic molecules account for ca. 20 wt.% [85]. Intrinsic tooth color is due to enamel light absorption and scattering; as HA is white, natural enamel has a white color with partial translucency [86]. On the other hand, the higher organic matter content of dentin gives it a yellowish taint. The progressive wear of enamel with age makes it thinner and more translucent, allowing dentin to be more visible and darkening the tooth color [87]. Apart from this naturally occurring phenomenon, teeth can also change color due to intrinsic or extrinsic stains. Intrinsic stains take place inside the tooth, either in enamel or in dentin. Commonly, these stains are associated to pathologies such as fluorosis (excessive fluoride intake at a tender age) or are due to amalgam fillings or blood from pulpal hemorrhages that penetrate into dentinal tubules and cause a dark color [88,89]. Extrinsic stains are due to the adsorption of organic and inorganic chromophores onto enamel and exposed dentin, especially on rough surfaces that are difficult to clean, or are incorporated into calculus (a pathologic calcification formed by plaque), or into the bacterial biofilm, which is an aggregate of bacterial cells embedded within a matrix of extracellular polymeric substances [89]. Organic chromophores are colored molecules such as tannins or nicotine from red wine, coffee, smoke, or tea, while inorganic chromophores are colored metal ions [89,90]. Moreover, some oral care products, such as chlorhexidine or SnF_2_, can induce an indirect staining after long-term use [90].

Several products for tooth whitening exist nowadays on the market and can be divided into two categories: (i) bleaching agents and (ii) abrasives [88,91,92]. Bleaching agents are usually in the form of gels that contain hydrogen peroxide or carbamide peroxide, and their effect is to produce reactive oxygen radicals that oxidize the chromophores, make them colorless and detach them from the tooth’s surface [93]. Abrasives are insoluble mineral ingredients, usually included in toothpastes, that remove the extrinsic stains by mechanical abrasion [94]. Common abrasives are silica, calcium carbonates, alumina, and perlite [95]. Abrasives were shown not only to remove stains, but also to prevent stain formation by polishing the teeth and thus forming a smooth surface that adsorbs less chromophores [96].

Whitening agents present drawbacks. The strong oxidation of bleaching agents degrades the organic matrix of enamel and dentin, while their acidic components damage the mineral component, creating porosities, grooves, and micro-cracks in the enamel, reducing its mechanical resistance, and causing a higher susceptibility to deformation and fracture [97,98,99]. Furthermore, the increased roughness of bleached enamel makes it more prone to new extrinsic staining and/or biofilm adhesion [100,101]. In addition, after bleaching, people commonly suffer tooth sensitivity due to opened dentinal tubules [102]. Finally, bleaching agents can also damage the bond between resin composites and dental hard tissues, making them more at risk of failure [103,104]. Regarding abrasives, their action is limited by the accessibility of the toothbrush to stained areas of the teeth, and less accessible sites can remain stained [92]. Furthermore, the prolonged use of very hard abrasives can give a significant wear of enamel and dentin [105,106]. Therefore, there is the need of innovative whitening agents to overcome the limitations of current materials. CaPs can fill this role, and several research works have already proved this [107,108,109,110,111,112,113,114,115,116,117,118]. CaPs as whitening agents have been proposed as innovative abrasive agents, as carrier of peroxides, or as adjuvant of bleaching agents.

**Figure 5:**
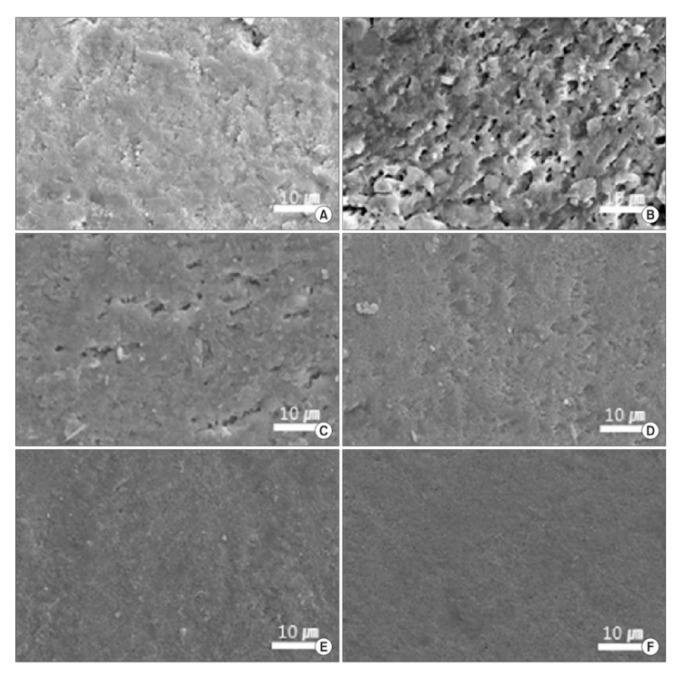
Scanning electron microscopy (SEM) micrographs of enamel surface treated with (**A**): control; (**B**): bleaching with 10% carbamide peroxide; (**C**–**F**): whitening treatment with different mixtures of HA, ACP, and tetrasodium pyrophosphate (TSP); (**C**): ACP 0.3%, HA 0.2%, and TSP 0.1%; (**D**): ACP 0.75%, HA 0.5%, and TSP 0.25%; (**E**): ACP 1.5%, HA 1.0%, and TSP 0.5%; (**F**): ACP 3.0%, HA 2.0%, and TSP 1.0%. (Reprinted from [115]).

In this regard, the distinction of CaPs between cosmetic ingredients and therapeutic ingredients is labile because CaPs have a two-fold whitening action: (i) as an abrasive, to remove the stains by mechanical friction, and (ii) as a remineralizing ingredient, to restore the enamel structure and improve light scattering and absorption, leading to teeth brightness and whiteness [109,112,116]. In addition, it was demonstrated that the remineralization action of CaPs makes the enamel surface smoother and less prone to staining [113,116]. The whitening effect of CaPs has been studied in vitro [116], ex vivo [109,110,111,112,113,114,115], and in vivo [107,108,115,116]. The majority of the works employ HA as CaP phase [107,108,109,110,111,112,113,114,115,116], as HA is the natural mineral phase of the teeth, while some other works also investigated the effect of TCP [109,111], or mixtures of HA, ACP, and pyrophosphates [113]. However, based on the published papers, it is not possible to assess whether a CaP phase is more efficient than another as a whitening agent. In addition, these works have studied the whitening effect of CaPs as a raw material [109,110,113,115], as a prototype gel or toothpaste formulation [112,116], or as a commercial product [107,108,111,114], making it very difficult to discriminate the influence of HA on the whitening effect from other ingredients. Furthermore, the analysis of tooth color is problematic because in vivo assessment is subjective and made by comparison with a color scale, while ex vivo is strongly dependent on the variability of the tested teeth [119]. Taking into account these aspects, it is not possible to compare the results between different works. The work of Niwa et al. [116] dated back to the early 2000s is the first to propose the use of HA as a whitening agent. In this work, the abrasiveness of toothpastes containing HA onto sintered HA blocks that mimicked enamel was studied, and the brightness and color of the teeth of people that used the toothpastes for 4 weeks were monitored. It was found that the wear caused by HA toothpastes was limited, while a significant increase in brightness and whiteness occurred, with the effect being dose-dependent on HA content in the product. From these results, it was postulated that the whitening effect of HA (and other CaP phases) is mainly due to the remineralization of enamel than by its abrasion. In this respect, the study of Dabanoglu et al. [109] has shown that HA or TCP particles attach to the enamel surface and are not removed even by the shear forces of brushing. This induced an improvement of color that increased at every treatment, becoming appreciable also with the naked eye after more than four treatments. The concept was further deepened by Lee et al. [113], who compared the whitening effect, surface attachment, and enamel remineralization of a commercial carbamide peroxide bleaching gel with those of a remineralizing gel containing three non-bleaching ingredients: HA, ACP, and tetrasodium pyrophosphate (TSP). The tested gels had a concentration of active ingredients that ranged from ACP 3.0%, HA 2.0%, and TSP 1.0% to ACP 0.3%, HA 0.2%, and TSP 0.1%. It was found that both the bleaching and the remineralizing gel generate a significant improvement of whiteness in comparison to the control, and that the remineralizing gel with the highest content of HA, ACP, and sodium pyrophosphate has an efficacy that was comparable to the carbamide peroxide gel. On the other hand, the bleaching gel induced a strong demineralization of enamel both on the surface and in depth (up to 60 μm), while the remineralizing gel restored enamel and improved its smoothness (Figure 5). Unfortunately, in this work the single role of HA, ACP, and sodium pyrophosphate was not studied and therefore it is not possible to assess the contribution brought by every single ingredient to the final effect.

**Figure 6:**
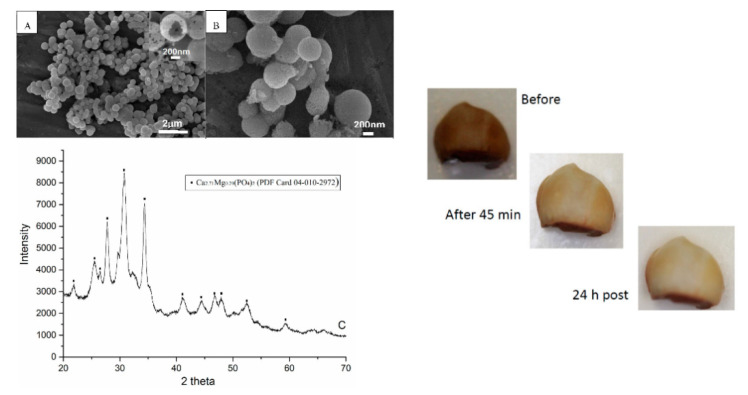
**Left**: (**A**,**B**) Scanning electron microscopy (SEM) micrographs and (**C**) powder X-ray diffraction (PXRD) patterns of TCP microspheres. **Right**: ex vivo bleaching effect of the microspheres containing hydrogen peroxide and carbamide peroxide. (Reprinted from [109]).

In the works of Mellgen et al. and Qin et al. [117,118], CaP microspheres were loaded with hydrogen peroxide and/or carbamide peroxide. The CaP microspheres were prepared by a hydrothermal process in the presence of strontium and magnesium ions and were proved to be spherical hollow particles with a diameter between 500 and 1000 nm constituted by Mg-doped TCP. These spheres adsorbed ca. 15 wt.% of carbamide peroxide (the amount of adsorbed hydrogen peroxide is not disclosed) and were found to slowly release it when incorporated into a gel formulation, inducing the whitening of the ex vivo tooth (Figure 6). The authors claim that this formulation has the advantage of having a sustained release of peroxide on the tooth’s surface over time in comparison to commercial bleaching agents due to the attachment of the microspheres on the enamel surface. At the same time, the microspheres could also be able to remineralize the structural damage caused by the bleaching.

CaPs have also found successful application as an adjuvant of common bleaching agents [120,121,122,123,124]. In this case, CaPs have an indirect action, as the whitening is generated by the bleaching ingredients and the purpose of CaPs is to remineralize the enamel damaged by the bleaching. The majority of works reporting on this application of CaP ingredients employ casein-phosphopeptide-stabilized ACP (CPP-ACP) [120,121,122,124], as this material is already commercially available and was proved to have a strong remineralizing action [59], even though in one study HA was also used [123].

Finally, all the articles on this topic proved that the presence of CaPs did not hinder the whitening action of the bleaching agents but allowed such agents to overcome their drawbacks. Indeed, the combination of CaPs with bleaching agents improved tooth whiteness and at the same time prevented enamel demineralization, hardness loss, tooth sensitization, and surface roughening [120,121,122,123,124].

The excellent whitening effect of CaPs reported in scientific literature is reflected by the high number of patents comprising CaPs as abrasive and bleaching agents reported in Table 1. In general, about half of the patents protect toothpaste or gel formulation containing CaPs [125,126,127,128,129,130,131], while the other half protect CaPs as an ingredient for generic whitening products [132,133,134,135,136,137,138,139,140,141,142,143,144,145,146,147]. This indicates that there is an interest in the use of CaP both as a commercial product and as refined materials to be incorporated into other products.

Indeed, the use of CaP as tooth whiteners has the lion’s share, with 29 registered patents that comply with our search criteria. The majority of these patents have been deposited in the last decade and derive from cosmetics companies [125,126,127,128,129,130,132,133,134,135,136,137,138,139,140,141,142,143,144,145,146,147,148,149,150,151,152], while on the other hand for two patents the inventors belong to universities [131,153]. Similar to the scientific literature, almost all of these patents protect the use of CaPs as a whitener in virtue of their remineralizing and abrasive action [125,126,127,128,129,130,131,132,136,137,139,141,142,143,146,148,152], although many of them do not disclose whether the abrasive effect or the remineralizing one is more relevant. An interesting application is reported in a patent by Mectron SPA [137], where the CaP powder is used for the air-polishing of teeth. There are also several patents on the use of CaPs in synergy with bleaching agents [135,138,144,147,150,153]. In these patents, the bleaching action is exerted by peroxides [135,141,143,144] but also by enzymes [138,147,150,153], where this latter case was never reported in scientific papers.

Apart from these two categories, some patents propose a whitening effect through the formation of a CaP coating onto teeth by using varnishes or polymers [133,134,140,149]. In this case, the function of CaP is to impart a white color that should be as close as possible to the natural color of teeth. The coating-forming agents can be varnished as the copal resin (natural tree resin) [149], or polymers such as polyvinylpyrrolidone [133,134,140] and polyvinylpyrrolidone/vinyl acetate copolymers [140]. These ingredients are dissolved together with CaPs in a volatile solvent, and when the solvent evaporates, they form a film onto enamel with CaP particles embedded in it. Therefore, in these patents, the enamel is actually not restored but only varnished with a white coating.

It is worth noting that all patents specify HA as the only materials of the invention, except for three patents [132,141,143] claiming that any CaP phase is suitable for their invention. The prevalence of HA over the other CaP phases is explained by the fact that it is the same mineral phase of enamel, and thus has a better remineralizing action due to its biomimetism. Interestingly, the patent [133] protects a method to directly produce HA onto the tooth’s surface by using TCP and hydrogen phosphate ions. All patents about tooth whitening do not involve ion-doped HAs, even if in literature the effect of ion substitution on HA for oral care application has been extensively studied. In this regard, only the patent [126] mentions the use of a strontium-doped HA. The patent [151] is interesting because it reports the invention of a toothbrush bristle containing HA and antibacterial ions for both tooth whitening and anti-caries action. In comparison to literature, patents on CaPs for dental whitening focus more on the remineralizing and stain removal effect than on the use of bleaching agents, suggesting that the former approach could be more appealing.

**Table 1:** List of patents about calcium phosphates in oral care.

Ref	Title	Applicants	Publication Year	Description
[132]	Set for tooth bleaching	Lion Corp	2000	Product for tooth whitening by stain abrasion. CaP: HA, fluorine or carbonate doped HA, TCP, OCP
[133]	Manufacturing method of hydroxyl apatite	Nippon Zettoc Co., Ltd.	2001	Coating varnish for tooth whitening. CaP: TCP + hydrogen phosphate ions
[140]	Rapid temporary tooth whitening composition	Colgate Palmolive Co	2005	Adhesive for tooth whitening by HA adhesion. CaP: HA
[125]	Dentifrice composition	Sangi Co., Ltd.	2005	Toothpaste for tooth whitening. CaP: HA
[141]	Dental whitening compositions	Discus Dental LLC	2006	Product for tooth whitening by stain abrasion and for desensitization and remineralization. CaP: HA, TCP, OCP
[142]	Instant tooth whitening with silicone resin and silicone adhesive	Colgate-Palmolive Company	2006	Product for tooth whitening. CaP: HA
[143]	Dental Whitening Compositions	Discus Dental LLC	2008	Product for tooth whitening by stain abrasion and for desensitization and remineralization. CaP: HA, TCP, OCP
[131]	Pastes for improving tooth using micro hydroxyapatite powders	Pusan National University Cooperation Foundation Ryu Su Chak	2009	Toothpaste for tooth whitening. CaP: HA
[148]	Oral composition	Sangi Co., Ltd.	2005	Product for tooth whitening and for remineralization. CaP: HA
[149]	Dental colorant	Zakrytoe Aktsionernoe Obshchestvo Opytno-ehksperimental’nyj Zavod “Vladmiva”	2011	Resin varnish for tooth dyeing. CaP: HA
[144]	Teeth whitening composition and method	OCSLabo—Oral Care Science Lab Sagl	2011	Product for tooth whitening by bleaching. CaP: HA
[145]	Compositions and methods for altering the color of teeth	Colgate Palmolive Co	2007	Product for tooth whitening by coating. CaP: HA
[126]	Total effect toothpaste and preparation method thereof	Yiwu Aishang Commodity Co., LTD.	2011	Toothpaste for tooth whitening. CaP: strontium doped HA, TCP
[150]	Mineral-enzyme complex for fortifying and whitening tooth enamel, oral hygiene composition and toothpaste	Belous, Elena Yurievna; Galimova, Anna Zufarovna; Maltabar, Svetlana Alekseevna; Obshchstvo S Ogranichennoj Otvetstvennostyu “Splat-Kosmetika”	2014	Product for tooth whitening by bleaching and for remineralization. CaP: HA
[151]	Brush structure with health-care effect	Nakata Tomoko; Tanaka Fumiko; Tanaka Kimiko	2014	Toothbrush bristle with whitening and antibacterial ingredients. CaP: HA
[146]	Tooth-whitening compositions comprising silicone polymer and methods therefor	Colgate Palmolive Co	2005, 2014	Product for tooth whitening. CaP: HA
[127]	Toothpaste for simultaneously cleaning, whitening, and restoring teeth and preparation method of toothpaste	Masson Group Co., Ltd.	2013	Toothpaste for tooth whitening by abrasion and remineralization. CaP: HA
[128]	Dentifrice composition comprising sintered hydroxyapatite	Glaxo Group Limited	2015	Toothpaste for tooth whitening by stain abrasion. CaP: HA (sintered)
[152]	Natural bacteriostatic tooth whitening powder and preparation method thereof	Qingdao Bright Medicine Hall Medical Treatment Co., Ltd.	2016	Powder for tooth whitening by stain abrasion. CaP: HA
[153]	Whitening gel composition based on natural agents	Universitatea “Babes Bolyai”—Institutul de cercetari in chimie “Raluca Ripan” Cluj-Napoca	2017	Gel for tooth whitening by bleaching. CaP: HA
[147]	Mineral-enzyme complex for strengthening and whitening tooth enamel, oral hygiene composition, and toothpaste	Elena Yurievna Belous, Svetlana Alekseevna Maltabar, Anna Zufarovna Galimova	2017	Product for tooth whitening by bleaching and for remineralization. CaP: HA
[134]	Tooth coating agent and compositions thereof	Medice Co., Ltd.	2018	Product for tooth whitening by coating. CaP: HA
[135]	Tooth cold-light whitening composition and application thereof	Jilin dengtaike Dentistry material Co., Ltd.	2018	Product for tooth whitening by bleaching and for remineralization. CaP: HA
[129]	Whitening toothpaste	Foshan Yuan Po Xin Technology Co., Ltd.	2018	Toothpaste for tooth whitening. CaP: HA
[130]	Whitening tooth paste capable of effectively removing stains on teeth and preparation method thereof	Anhui Wanchun Daily Chemical Co., Ltd.	2019	Toothpaste for tooth whitening. CaP: HA
[136]	Dental care product for tooth whitening	Credentis AG	2016	Product for tooth whitening by stain abrasion and for desensitization and remineralization. CaP: HA
[137]	Method for teeth cleaning by means of a composition in the form of powder based on hydroxyapatite	Mectron SPA	2019	Product for tooth whitening by air polishing. CaP: silica particles containing HA
[138]	Mineral-enzyme complex for strengthening and whitening tooth enamel, oral hygiene composition, and toothpaste	Obshchestvo S Ogranichennoj Otvetstvennostyu “Splat-Kosmeticka”	2014	Product for tooth whitening by bleaching and for remineralization. CaP: HA
[139]	Pearl whitening and refreshing toothpaste and preparation method thereof	Henan Shuiantinglan Cosmetics Co., Ltd.	2020	Product for tooth whitening by stain abrasion. CaP: HA

### 3.2. Skin Care

The skin is the largest body organ, and its key role consists of protection against several harmful environmental agents, such as pathogens, chemical threats, radiations, temperature changes and dehydration. The skin is composed of several components, acting as (i) a physical barrier, which consists of the stratum corneum, (ii) a chemical or biochemical barrier that consists of fatty acids, antimicrobial peptides, and macrophages, and (iii) an immunological barrier composed by humoral and cellular constituents of the immune system. The stratum corneum serves as the principal barrier against percutaneous penetration of chemicals and microbes and is capable of strong mechanical forces. Furthermore, it is involved in the transepidermal water loss, consisting of the regulation of the water loss from the body to the surrounding atmosphere via diffusion and evaporation processes [154,155,156]. To ensure excellent skin conditions, it is necessary to consider, control, and optimize several skin parameters, such as surface texture, color, and physiologic properties (hydration, sebum content, and surface acidity). Furthermore, it was proved that there are also interactions between skin state, diet, and the content of nutrients in blood serum. Indeed, Boelsma et al. reported that changes in diet and intake of nutrients may affect skin conditions [157]. In general, macro and micronutrients such as vitamins or minerals have a relevant importance for skin health and appearance [158,159,160]. For this reason, micronutrients are used as therapeutic agents for skin diseases and as ingredients in cosmetics, which nowadays are designed not only to beautify the skin, but also to improve skin health [161,162,163]. The beautification of skin is referred to as make-up, while the improvement of skin health is achieved by skin care routines (e.g., daily cleaning, scrubbing, and hydration). Most of the works in this field—which will be discussed in the present section—are focused on the use of CaPs as: (i) sunscreens, i.e., products that protect from solar UV radiation, (ii) cleansers, which are products that ensure skin health by removing sebum or contaminants and promoting normal exfoliation, and (iii) make-up products, which beautify the skin and hide topical disorders.

A total of 61 patents focused on CaPs in skincare cosmetics were deposited between 2001 and 2021. These are divided as follows: 10 patents on sunscreens, 17 on skin cleansers, and 30 on make-up formulations. However, there are four patents on the use of CaPs as skin care material that do not fall into any of the above categories and are very different from each other (Table 2) [164,165,166,167]. Two of them employ CaPs as a vehicle to deliver nutraceuticals to the skin [165,167], as CaPs are widely known to be efficient carriers to deliver biomolecules into cells and tissues. Contrastingly, in patent [164], HA is used to stabilize and deliver the ubiquinone molecule (coenzyme Q10), a nutrient widely used in cosmetics that suffers of poor thermal stability. In the majority of these patents, the full chemical composition of the materials is not disclosed and generally are organic–inorganic hybrids of CaP and polymers, such as HA and polyhydroxyalkanoates [165], HA and polycarbonates [167], and CaPs and poly(L-dihydroxyphenylalanine) [166].

#### 3.2.1. Skin Protection—Sunscreen (UV Protection)

Natural sunlight consists of a wide spectrum of electromagnetic waves. The solar ultraviolet (UV) radiation that reaches the earth is composed of 5–10% of energetic UVB (290–320 nm) radiation and 90–95% of UVA (320–400 nm). The latter is less energetic than UVB, but it penetrates deeper into the skin due to its longer wavelength. The exposure to solar radiation, especially UVA and UVB, can cause skin diseases such as sunburn, erythema, photoaging, wrinkles, altered pigmentation, or even skin cancer [168,169]. To prevent these diseases in cases of strong or prolonged sun exposure, it is necessary to use sunscreens to protect the skin and to reduce the risks of melanoma and other types of cancer [170,171]. As reported by Pirotta in the book of Tovar-Sanchèz et al. [172], “sunscreens reflect, absorb, and scatter both UVA and UVB to provide protection against both types of radiation”. Usually, commercial sunscreens contain different UV filters to obtain a formulation that is effective at protecting from the whole UV range. Serpone et al. and Egambaram et al. published two review papers that resume a discussion of the most common topical sunscreen agents used nowadays and consider such agents’ effect on skin, the current concerns on their health and environment safety, and future perspectives in the sunscreen field [173,174]. Generally, UV filters are categorized in two classes: (i) organic or chemical filters, capable of absorbing UV radiation, and (ii) inorganic or physical filters, which reflect or scatter incident radiation. Both kinds of UV filters can cause problems for one’s health and the environment, which are widely reported [175,176,177,178]. Indeed, filters might cause photo-allergies, phototoxic reactions, and skin irritations. Moreover, the high refractive index of inorganic filters decreases the aesthetic value of sunscreens due to the unnatural white color acquired by the skin after application [179,180]. To solve these drawbacks, inorganic filters are frequently used in a micro- or even nano-sized form, but this latter form might cause the penetration of the UV filter into the skin, causing skin allergies or irritation [181,182].

Titanium dioxide (TiO_2_) and zinc oxide (ZnO) are the most used inorganic UV filters. These filters can be particularly dangerous when they are in nano-sized form due to their photocatalytic properties. Indeed, under sunlight irradiation, the photocatalytic process generates free radicals or other reactive species that could cause skin damage or long-term illnesses, and this can occur even if these NPs are coated by an inert oxide layer [183,184,185,186,187,188,189,190]. However, the discussion about the dangerousness of these inorganic UV filters is still open, as despite the recent concerns on their safety, TiO_2_ and ZnO were proposed by the FDA as the only two Generally Recognized As Safe and Effective (GRASE) ingredients for sunscreens, prompting the need of more research on the topic of percutaneous absorption and the health effects of UV filters [191].

Regarding organic filters, a recent review by Narla et al. about the last FDA results confirms the high danger level of some filters for both humans and the environment [192]. Several studies show that UV filters contaminate almost all water sources around the world, and the removal of these substances by using common wastewater treatment techniques is very difficult [176,193]. Sunscreen pollution in a marine environment brings serious consequences in a coastal ecosystem. It was estimated that 14,000 tons of UV filters are released in coral reef areas each year, leading to the coral bleaching phenomenon, a consequence of UV filter toxicity that causes the death of the coral organism by photo oxidation with the production of reactive oxygen species (ROS), by endocrine disruption and by DNA damage [194].

To overcome these problems, alternative and effective sunscreens should be developed. The requisites of the ideal sunscreen should be broad protection over UVA and UVB, non-toxicity, no photocatalytic effects, and environmental safety. In recent years, HA has been proposed as a safe sunscreen ingredient, and the interest in CaPs for this application is growing [195,196]. In the literature, there are several examples that report the preparation of HA as a safe inorganic sunscreen ingredient to replace ZnO and TiO_2_ [197,198,199,200,201,202,203,204,205,206,207,208,209,210,211]. Indeed, it was found that HA shows not only non-toxicity and biocompatibility properties, but also screening capability, high dermal tolerance, and a lower whitening effect than other inorganic sunscreen agents. HA can be either synthetic or can be obtained from natural sources. In the next sections, the use of synthetic or natural HA as a sunscreen ingredient will be described. However, the main problem of both synthetic and natural CaPs for sunscreen application consists in their intrinsic UV absorption limit, which is related to the electronic structure. In fact, CaP has optical absorption only in the range from 200 to 340 nm with a strong band below 247 nm, and it depends on the thermal treatment carried out on it [212], but by introducing foreign elements into the CaP crystal structure as dopants it is possible to improve their limits in the adsorption. The addition of metal cations into the HA structure is allowed by a cation exchange reaction with the calcium ions on the surface of the material. In the literature, there are many articles that describe the differences between the absorption spectrum of doped and undoped CaPs, allowing the material to absorb in the range from UVB to UVA. In the next sections, several examples regarding the advantages of synthetic and natural HA also doped with other elements or transition metals to improve UV protection will be described.

##### Synthetic Calcium Phosphates as Sunscreens

As discussed thoroughly before, one of the major advantages of CaPs consists in the easy possibility of controlling and tuning the chemical and physical properties varying the synthesis parameters, thus making them suitable as sunscreen ingredients [213]. In their research, Amin et al. studied a sunscreen system based on rod-like nanosized monoclinic crystalline HA modified by ascorbic acid (AA) and stabilized with poly(vinylpolypyrrolidone) (PVP). This work aimed to prepare an advanced sunscreen that harnesses the UV optical absorption of HA and AA, and at the same time is able to scavenge the dangerous ROS due to the presence of AA [197]. The authors observed a significant decrease in the level of intracellular ROS of cells treated with a different concentration of the nanocomposite. Moreover, this material showed absorption peaks at 225 and 250 nm that represent the blue shift of the absorption of pure HA at 247 nm and of AA at 262 nm, respectively. The blue shift confirmed the correct conjugation between HA, AA and PVP. Morsy et al., instead, developed a multifunctional hydroxyapatite–chitosan (HA–chitosan) gel that works as an antibacterial sunscreen [198]. Despite the capability of protecting the skin from UV exposure due to its reflectivity and good opacity, HA does not show any antibacterial activity. To prevent side effects such as erythema, blisters, and pimples caused by the solar UV radiation, this work proposed the use of chitosan as an anti-bacterial natural compound. They noticed that the multifunctional gel exhibited optical absorption in the ultraviolet (254 nm) and showed a significant inhibitory effect on the growth of multidrug-resistant bacterial colonies.

Finally, to improve the UV absorption of HA-based materials, a cation exchange between calcium and other transition or metallic elements has been proposed. An example is Fe^3+^ that is largely used as dopant in HA and, as other elements, it could lead to an HA lattice contraction or extension depending on the iron concentration and on the mechanism of iron insertion [214,215]. In addition, as a result of the exchange between Fe^3+^ and Ca^2+^ the Ca/P ratio decreases showing the formation of a calcium-deficient HA [201]. The addition of Fe in HA improves the optical properties of the material; in particular, it shows a higher UV absorption over the whole UV range [202,203,204]. In addition to iron, in the literature are reported articles in which the influence of Mn^2+^ and Zn^2+^ in the photo-physical properties of HA using different methods of production are studied. De Araujo et al. evaluated the optical properties of HA or β-TCP doped with these latter ions, but also the effect of Cr^3+^, Zn^2+^, and Fe^3+^ in the HA structure [206,207]. The authors evaluated the optical absorption of pure β-TCP and β-TCP doped with Zn^2+^ and Mn^2+^, and they concluded that β-TCP cannot be used as an ingredient in sunscreens because it did not absorb in any region of the solar spectrum. They observed that the introduction of Cr^3+^, Fe^3+^, Zn^2+^ and Mn^2+^ generates different absorption bands in comparison to pure HA, which absorption band presents a maximum at 207 nm. In particular, the addition of Fe^3+^ and Cr^3+^ allowed for the enlargement of the HA absorption spectrum from the UV to visible region, even though this could induce the generation of a colored film of sunscreen. Fortunately, this problem could be resolved by optimizing the cosmetic formulation and providing a good dispersion of the ingredient on the skin, giving a transparent appearance when applied. In summary, the authors reported the successful production of doped HA with suitable optical absorption that could be used as an active ingredient in sunscreens. The papers of De Araujo et al. have inspired more studies about the comparison between doped HA and pure HA structures for sunscreen applications [208,209]. Eventually, another element that was incorporated to modify the UV properties of HA is silver. In their research, Pyo et al. reported the preparation of silver-doped HA (Ag-HA) NPs and the study of their blocking capability of UV and visible radiation. They observed that the addition of Ag significantly increased both UV and visible absorption compared with pure HA [211]. Ag^+^ and Zn^2+^ are widely known as ions showing an antibacterial effect even when added to CaPs, but this feature was not evaluated in the papers about sunscreens reported above. Therefore, it is certainly of interest to further study the effect of CaPs doped with Ag^+^ and Zn^2+^ as a multifunctional material with antibacterial and sunscreen abilities.

##### Natural Calcium Phosphates as Sunscreens

Sunscreen is the only cosmetics sector where, according to scientific literature, CaPs from natural sources were also used. All of them come from fish industries, as they produce large amounts of by-products every year; therefore, the CaP production from fish by-products for different applications is widely studied because it has great relevance in terms of the benefit for both the environment and the economy of the industrial sector [65,216,217,218]. An example is reported by Hernandez-Cocoletzi et al. that developed a system based on HA achieved from fishbone waste in order to absorb heavy metals from aqueous effluents [219]. Granito et al. exploited the high potential of this kind of HA from natural sources in biomedicine application due to the intrinsic biocompatibility of the material [220]. Furthermore, Mohd Pu’ad et al. provided an interesting review about several methods to synthetize HA from natural sources, including the marine ones, which are: thermal treatment, alkaline hydrolysis, wet precipitation or mechanochemical processes [221]. Therefore, in recent years, the use of HA from marine sources as sunscreen materials has received increased interest [199,200,222]. Cunha et al. reported the preparation and characterization of films made of chitosan and Fe-modified HA of marine origin. They noticed excellent UV-absorbing properties, antibacterial activity, and non-cytotoxicity. This material could be used for wound dressing, because it reduces bacterial infection while protecting wounds from UV exposure [205]. Another example is the work of Ghazali et al., which reports the preparation of an active sunscreen ingredient from clamshells by substituting calcium ions of HA structure with Fe^3+^ or Mn^2+^ [210]. The authors characterized the materials through X-ray diffraction, infra-red spectroscopy, and UV-visible spectrometry, and they determined the capability of samples to absorb UV light. They noticed that the substitution of calcium ion with Fe^3+^ and Mn^2+^ increased the absorption values of pure HA because these dopants can reflect UV light. Therefore, they highlighted an increase in the sun protection factor (SPF) of emulsions prepared with Fe-HA and Mn-HA compared to the emulsion of pure HA.

##### Patents about Calcium Phosphates as Sunscreens

Overall, the use of CaP as an ingredient in sunscreen products has recently gained interest, as shown also by the production of patents. Indeed, there are 10 deposited patents about CaP products with a sunscreen effect, and most of them were deposited in the last 5 years (Table 3). All patents protect the preparation of a raw ingredient that adsorbs or scatters UV radiation and can be incorporated into sunscreen formulations [223,224,225,226,227,228,229,230,231,232]. In some cases, CaPs are directly responsible for UV shielding [223,224,225,227,228,229,230,231], while in other cases their function is to boost the efficiency of an organic filter [226,232]. In comparison to other patents described herein, for sunscreen application there is a wide variety of CaP phases: HA [224,226], ion-doped HA [228,229,230,231], mixtures of HA and metal oxides [228,230,231], composites of HA with organic molecules [232] or with inorganic materials [223,225], and in some cases the mineral phase was not reported at all [227,232]. This variety suggests that for this application the crystal phase of CaP is not critical, while chemical composition has a higher relevance. Indeed, as also reported in the literature, in a high number of patents the UV absorption of HA was enhanced by incorporating iron or titanium ions into its structure—even if the patents do not exclude the formation of metal oxides as side products [228,229,230,231], or by associating HA with UV-adsorbing inorganic or organic materials [223,225,232]. It is worth mentioning the patent [231], in which the CaP material derives from renewable sources, specifically fishbones. Overall, the approaches used in the patents described so far are similar to those reported in scientific literature ((i) the use of ionic doping with transition metals and (ii) the use of CaP from natural sources). The use of CaPs as an SPF-booster of organic UV filters has been reported in three patents, including the two most recent ones [226,231,232]. An SPF-booster is an inorganic material that enhances the absorbing properties of organic filters by scattering the incident UV radiation. The use of CaPs as boosters is one of the most interesting approaches in their application for sun care cosmetics.

#### 3.2.2. Skin Cleaners

As mentioned before, the skin is an important organ that protects us from the environment. For this reason, it is necessary to ensure the skin health also by using cleansers, which are surfactants used to reduce sebum, remove make-up residuals or exogenous contaminants such as sweat, pollution or dirt, and promote normal exfoliation [233,234,235,236,237]. However, the repeated and prolonged interaction between these surfactants and the proteins or lipids of stratum corneum could weaken the skin barrier function. The consequences of skin barrier degradation might be the after-wash tightness, which consists in rapid evaporation of water from the skin surface causing tightness, itch, dryness, irritation, or inflammation [238,239]. Therefore, there is a need to develop cleansing systems that preserve and respect the skin barrier. The colloidal stability of cleansers is critical and thus the use of stabilizing agents is necessary. In this regard, scientific literature reports considerable examples describing the advantages of using solid particles (Pickering emulsions) [240,241] or natural minerals to achieve the optimal emulsion stabilization for topical application purposes [242,243,244]. In recent years, the use of solid particles was largely studied because they might offer sustainable and eco-friendly solutions as stabilizers. Among all the solid particles, calcium carbonate is often used for this purpose [245,246,247]. An example is reported by Marto et al. that has optimized a biocompatible Pickering emulsion formulation using calcium carbonate as a stabilizer [248]. Regarding CaP, to the best of our knowledge, there are no articles that describe these materials as stabilizers even if they could successfully substitute calcium carbonate in Pickering emulsions.

Contrastingly, skin cleansing is actually a well explored cosmetic application of CaPs by the industry, with 17 published patents, even though the majority of them were deposited more than 10 years ago (Table 4) [249,250,251,252,253,254,255,256,257,258,259,260,261,262,263,264,265]. Almost all patents harness the high adsorption capability of CaPs to adsorb sebum [252,253,254,255,256,258,259,260,261,264], while the others use CaPs to promote skin turnover or generically clean the skin [249,250,251,257,260,261,262,263,265]. For the former application, the role of CaPs is to adsorb and hold sebum, and in particular the fatty acids of sebum, in order to improve skin cleanliness, to favor make-up product adhesion and avoid make-up smearing, and to prevent the formation of malodorous substances. In the case of generic skin-cleaning patents, the role of CaPs is to favor the exfoliation of dead skin though their abrasive action, as well as removing dirt and bacteria. Interestingly, all the selected cleansing patents protect only complete formulations, in contrast to the patents about CaPs for other cosmetic applications [249,250,251,252,253,254,255,256,257,258,259,260]. It is likely that the cleansing effect arises from the whole combination of the ingredients of the formulation, and for this reason the whole final product was protected. Similarly to the patents for oral and sun care, the majority of skin cleansing patent refers to HA as the CaP phase [249,251,252,253,254,255,256,259], while only in few cases other phases as TCP, OCP, pyrophosphates, and ACP, are reported [257,258]. In the patent [250] the term “amorphous HA” is used, which is incorrect since HA is a crystalline phase, and it likely meant an HA with poor crystallinity. While in most of the patents HA was used as it is, in others it was used as a coating of inorganic or polymeric materials [255,256,258,259,263]. It is interesting to note that there is a mismatch between the high number of patents on CaPs as skin cleansing agents and the complete absence of scientific papers on the same topic. A possible explanation for this is that skin-cleaning formulations, being very complex and rich in coadjutants, are difficult to study in a systematic way since the individual effect and influence of each ingredient cannot be extrapolated.

#### 3.2.3. Skin Beautifying—(Make-Up)

The use of cosmetics for beautification has increased due to the desire to achieve high ideals of beauty imposed by modern society. Make-up could be included in the group of beauty treatments, which have a positive impact on people. Studies have revealed that the use of make-up products improves the well-being and affects the self-confidence of people [266,267,268]. Furthermore, another use of make-up consists in hiding topical disorders. An example is the bleaching of dark spots, also called age spots, characteristics of hyperpigmentation, demanding skin-whitening products [269,270]. The repeated contact between make-up products and skin may expose consumers to both localized skin problems and systemic effects caused by the absorption of chemical substances. In particular, the ingredients of make-up products derived from mineral pigments used as coloring agents could contain several harmful elements due to their origin or preparation process (i.e., Sb, As, Cd, Cr, Co, and Ni) [3,11,271].

Finally, cosmetic ingredients for make-up are commonly used in powder form to provide adhesiveness, smoothness, absorbency, and coverage. Moreover, powder ingredients can also be found in liquid formulations, where they improve cohesion, viscosity, or texture. The use of mineral powders (talc, silica, mica, starches, or clays) lead to a matte make-up product, but at high concentrations they show a lack of luminosity and may exhibit poor color stability [272]. Among all the powder ingredients, talc represents the most controversial ingredient. Indeed, its safety has been the topic of lots of debate over the years [273,274,275]. Recently, Fiume et al. assessed that the use of talc could lead to granulomas if it is applied on the skin when the epidermal barrier is absent. Therefore, it is necessary to find novel powder ingredients that are safe and biocompatible even if the skin barrier is damaged [276]. For this purpose, Bamford et al. developed a mesoporous magnesium carbonate (MMC) material with a high surface area and pore volume that can be used as a powder ingredient [277]. The authors noticed that the MMC shows an excellent absorption capacity, provides a long-lasting mattifying effect, and does not induce any skin irritation or sensitization. By similarity, another class of materials that could be used as a powder ingredient in cosmetic formulations is CaP, which may improve the biocompatibility of the final product while providing the same effects. Currently, there are no papers that describe these materials as a possible biocompatible substitution to controversial powder ingredients such as talc. On the other hand, 30 patents on this topic were filed over the years (Table 5). In particular, there are patents on make-up stabilizers and anti-smearing agents [278,279,280,281,282,283,284,285], foundations [223,279,286,287,288,289,290,291], pigments [281,287,292,293], lipsticks [294,295], products for enhancing skin collagen fibers [296,297,298], and many others. Several patents protect CaPs as enhancers for conventional make-up products, imparting an anti-sebum effect due to their adsorption action, giving a smooth sensation on the skin related to their microstructure, and incrementing make-up persistence and resistance to sweat and sebum. In this regard many different CaPs were patented, as HA [223,279,280,282,284,287,288,289], composites of HA or other CaP phases and inorganic particles [223,279,284,287,288,289,299], or composites of HA with organic particles [280,282].

Another interesting application of CaPs is in cosmetic pigments. In this case, the function of CaP is to impart a white color [292], or to host colored cerium phosphors [293], or to stabilize oil-soluble dyes [281,287]. For the first and last application, HA was the chosen crystal phase. Additionally, CaPs were also introduced in anti-age products whose aim is to stimulate production and to restore skin collagen fibers, incrementing skin elasticity [296,297,298]. In this case, the claimed action of CaPs is to directly stimulate skin fibroblasts to produce new collagen. The patent [300] is particularly interesting, as it protects the use of HA NPs as a stabilizer for O/W Pickering emulsion. As for other cosmetic patents, almost all patents about CaPs for make-up claim the use of HA as the preferred crystal phase, while some others employ mixtures or composites of CaPs with ZnO, TiO_2_, or other inorganic materials [223,279,286,287,289,299]. It is interesting to note that contrastingly to the other applications, ion doping was not considered to improve CaP properties. Even if the high number of patents suggests that there is interest in this field, there is no peer-reviewed data on the efficacy of these formulations or accepted standards on material testing for this application.

**Table 5:** List of patents about calcium phosphates in make-up.

Ref	Title	Applicants	Publication Year	Description
[286]	Powder cosmetics	Kose Corp	2004	Cosmetics powder product to be loaded on sponges and mats. CaP: HA and zinc oxide on a flaky powder
[278]	Cosmetics paper	Shiseido Co., Ltd.	2002	Sebum absorbing paper. CaP: HA
[279]	Solid powder cosmetics	Kose Corp	2005	Cosmetics powder product with make-up persistence and UV shielding. CaP: HA as sandwich between zinc oxide and platy powder
[297]	Therapeutic calcium phosphate particles in use for aesthetic or cosmetics medicine, and methods of manufacture and use	Biosante Pharmaceuticals, Inc.	2006	Anti-age cosmetics for topical application. CaP: non-disclosed
[223]	Coated powdery material and cosmetics containing the same	Pola Chem Ind INC	2006	Powder material for make-up of UV-shielding product. CaP: HA as layer with titanium oxide, alumina, silica
[295]	Stick-line cosmetics	Shiseido Co., Ltd.	2007	Stick-like cosmetics for lips. CaP: HA
[287]	Makeup cosmetics	Club Cosmetics Co., Ltd., Sekisui Plastics Co., Ltd.,	2007	Make-up cosmetics product. CaP: ACP-coated glass flakes
[288]	Powdery cosmetics	Shiseido Co., Ltd.	2008	Cosmetics powder product as foundation or make-up base. CaP: HA or HA composites
[289]	Solid powder cosmetics	Pola Chem Ind INC	2008	Cosmetics powder product. CaP: HA coating on sericite
[280]	Cosmetics	Clover Cosmake: Kk, Sekisui Plastics Co., Ltd.	2009	Product for suppressing smearing of make-up. CaP: dicalcium phosphate mixed with resin particles
[294]	Cosmetics composition for lips	Amorepacific Corporation	2010	Cosmetics product for lips. CaP: HA
[298]	Topical formulations comprising hydroxyapatite particles for stimulation and maintenance of collagen fibers	Laboratory Skin Care, Inc.	2010	Product for stimulation and maintenance of collagen fibers of skin. CaP: HA (sintered)
[281]	Bright pigment and cosmetics composition using the same	Nippon Sheet Glass Co., Ltd.	2010	Bright pigment and stabilizer for make-up. CaP: HA
[296]	Collagen production enhancer	SofSera Corp	2012	Product for stimulation and maintenance of collagen fibers of skin. CaP: HA
[301]	Two-in-one mixed curative effect type cosmetics	Zhou Qinghai	2013	Whitening and freckle-removing product. CaP: not disclosed
[290]	Cosmetics material and cosmetics	Horie Kako Co., Ltd.; Kinki University; Sofusera Co., Ltd.	2015	Cream or make-up cosmetic products. CaP: HA (sintered)
[282]	Composite particle and a cosmetics composition containing the same	Chanel Perfume Beauty Company	2015	Product for suppressing smearing of make-up and sebum adsorption. CaP: ACP coating of resin particles
[287]	Makeup cosmetics	Shiseido Co., Ltd.	2016	Make-up colored product. CaP: HA
[302]	Preparation method and application of fibroblast growth factor covering lipide calcium phosphate nanoparticles	Guangzhou Jipeng Biotechnology Co., Ltd., Medical and Biological Technology Research and Development Center Jinan Univ G	2016	Product for drug delivery to the skin. CaP: non-disclosed lipid-coated CaP nanoparticles
[299]	Calcium carbonate complex	Kotegawa Sangyo Kk	2017	Oil adsorbing product. CaP: HA coating of calcium carbonate
[291]	Makeup cosmetics	Shiseido Co., Ltd.	2018	Make-up colored product. CaP: HA
[300]	Stable O/W-type pickering emulsion by using hydroxyapatite nano particles and preparation method thereof	Xuchang University	2018	Pickering emulsion stabilizer. CaP: HA
[303]	Collagen production promoting agent	Sofsera Corp	2018	Product for stimulation and maintenance of collagen fibers of skin. CaP: HA (sintered)
[283]	Powder cosmetics and makeup method	Mikimoto Seiyaku KK	2018	Product for make-up stabilization. CaP: HA
[292]	Preparation method of whitening and moisturizing cream containing bismuth oxychloride	Guangzhou Lakel Stem Cell Research Institute	2020	Whitening moisturizing cream. CaP: HA
[293]	Calcium phosphate cerium phosphor	Sakai Chem Ind Co., Ltd.	2020	Phosphorescent cosmetics product. CaP: Non-disclosed cerium doped CaP
[284]	Cosmetics	Kose Corp	2020	Base material for cosmetics stabilization. CaP: HA-zinc oxide composite
[285]	Composition with makeup maintaining and oil controlling effects and cosmetics	Guangdong Bawei Biotechnology Co ltd	2020	Base material for make-up stabilization and sebum adsorption. CaP: HA

### 3.3. Hair Care

Hair does not have vital functions, but it represents an element of body image, and it is a complex organized structure that aims to protect the scalp. In particular, it is composed of proteins and different morphological components: (i) cuticle, consisting of several layers of thin and flat cells that aim to overlap one another to protect the cortex from physical and chemical insults; (ii) cortex, consisting of thick, rod-like cells, which contain keratin protein; and (iii) the medulla, which is located at the fiber’s center and consists of round cells, separated by air pockets [304,305]. Hair products can be categorized into two main categories on the basis of the duration of the treatment effect: (i) temporary products, such as shampoos, conditioners, sprays, and temporary dyes, and (ii) permanent products, such as permanent waves, relaxers, bleaches, and permanent dyes. In general, the repeated and systematic exposure to potentially harmful molecules contained in hair products can have negative outcomes [306,307,308,309,310]. An innovation in hair care products could be the use of NPs. As reported by Rosen et al., nanocarriers have optical transparency, due to their nanometric size, which enhances their cosmetic appeal [311]. Moreover, NPs are able to encapsulate insoluble ingredients, optimizing their delivery and their penetration into hair. For this reason, NPs were studied to specifically target the hair follicle and shaft, reintroducing necessary nutrients for the proper growth, texture, and health of the hair. To the best of our knowledge, there are no articles suggesting the use of CaP NPs in this field. However, considering several pieces of evidence in the literature that suggest the capability of CaP to act as a nanocarrier in the drug delivery system [22,42,312,313,314], this material can easily find application also in hair care products with the same purpose. Despite what has just been written and contrastingly to the other applications, the use of CaPs as hair care ingredients is not common.

Indeed, according to our search, only two relevant patents were found, where the application of CaP is as a dye carrier inside temporary hair dye products (Table 6) [315,316]. In both cases, HA was used thanks to its ability to encapsulate water-insoluble ingredients, optimizing their delivery and their penetration into hair. Interestingly, both patents report the use of HA as an ingredient for solid hair products. This is probably related to the rising interest in the development of solid shampoos as an eco-friendly alternative to liquid ones. Considering the continuous request for innovative, safe, sustainable and eco-friendly ingredients in cosmetics, it is expected that more patents and scientific papers on this topic will be published in the next years.

### 3.4. Deodorants

Body odor, especially axillary odor, is mainly generated by bacterial metabolism and oxidation of organic molecules of sweat. Sweat is produced by eccrine, apocrine and sebaceous glands and at the moment of its release is odorless, but the metabolism of cutaneous bacteria quickly transforms some of its components in several malodorous volatile substances, such as alcohols, aldehydes, ketones, and fatty acids [317,318]. The cosmetics industry has developed deodorant products in order to suppress or mask body odors. Deodorant ingredients can be divided into three categories on the basis of the method to control the odor: (i) antiperspirants, which suppress the production of sweat by physically blocking the glands duct, (ii) antimicrobials, which inhibit bacterial activity, and (iii) fragrances, which cover the malodors [319]. Usually, commercial products employ more deodorant ingredients in order to improve their effectiveness. However, even if these ingredients are widely used, they have also serious limitations. The most common antiperspirants are water-soluble aluminum chloridrate salts, which form aluminum hydroxide plugs inside the glands, that obstruct the excretory tubule, preventing sweating [320,321]. At the same time, these salts produce HCl as a side product, which causes skin irritation and erythema, and furthermore the aluminum metabolization can also cause toxicity effects [322,323]. The extensive use of antimicrobials, on the other hand, can alter the axillary microbiome, which might also favor the survival of odor-inducing bacteria [324,325].

**Figure 7:**
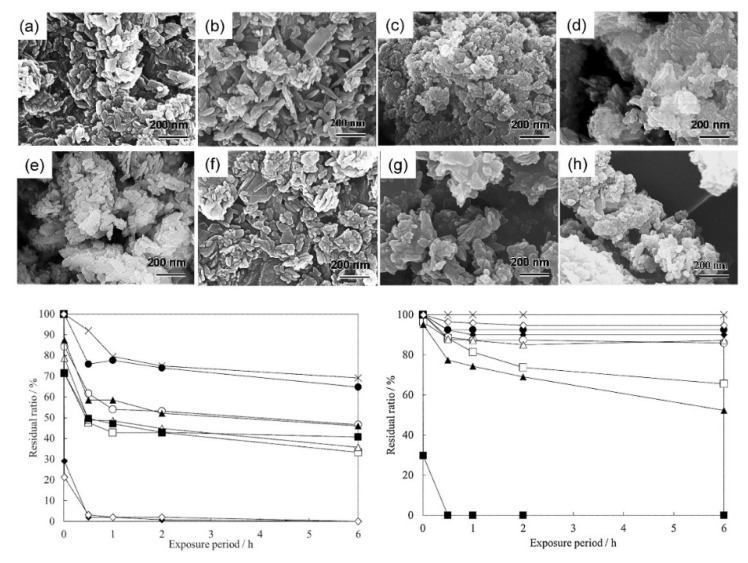
Scanning electron microscopy (SEM) micrographs of HA doped with (**a**) Ca, (**b**) Fe(II), (**c**) Fe(III), (**d**) Co, (**e**) Ni, (**f**) Cu, (**g**) Zn, and (**h**) Al. Below: adsorption of NH3 (lower left) or H_2_S (lower right) by HA doped with Ca (full circle), Co (empty circle), Zn (full triangle), Ni (open triangle), Cu (full square), Fe(II) (open square), Fe(III) (full diamond), and Al (open diamond). (©Elsevier. Reprinted with permission from [332]).

Recently a new category of deodorant ingredients, the adsorbing materials, has been proposed as an interesting alternative to the currently used ingredients to overcome their limitations. Adsorbing materials suppress malodor by adsorbing volatile malodorous substances through non-covalent interactions, and thus limit their volatilization. In this regard, CaPs can be interesting adsorbent materials for malodor control. Indeed, biomedical research has shown that CaPs can adsorb a wide variety of organic molecules, such as carboxylic acids, amino acids, proteins, nucleic acids, urea, and many others [326,327,328,329,330,331]. In addition, the presence of both positive and negative charges on the CaP surface allows for adsorbing both cationic and anionic molecules. CaPs can be also easily prepared as nanomaterials that, due to their high surface to volume ratio, have a high surface area and can adsorb great amounts of organic molecules. Despite these promising features, the use of CaP as an adsorbent for malodorous molecules is still in its infancy and the number of research articles on this application is scarce. In the work of Nishida et al. [332], the adsorption capability of pure and metal-doped HA toward malodorant H_2_S and NH_3_ gases was studied. HA was doped with divalent and trivalent cations (e.g., Co^2+^, Cu^2+^, Fe^2+^, Ni^2+^, Zn^2+^, Al^3+^, and Fe^3+^) by ionic exchange with surface Ca^2+^ ions to introduce the metal ions onto the HA particle surface. The adsorption efficiency of each gas on the various samples was related to surface composition, surface area, and the chemical nature of doping ions. It was found that NH_3_ was strongly adsorbed by HA doped with trivalent metals Fe^3+^ and Al^3+^, with less than 5% gas left after 30 min of exposure and was attributed to cation’s Lewis acidity and to structural effects (Figure 7).

In the case of H_2_S, only Cu^2+^-doped HA showed a strong adsorbing capacity, which was even higher than pure copper salts. It was hypothesized that the adsorption capacity of Cu-HA was related to the electronic structure of copper in the crystal lattice. In a follow-up work authored by Nishida et al. [333], a composite material of HA and zeolite was prepared and subsequently functionalized with Cu^2+^ and amino groups. It was found that the copper-doped composites have an excellent adsorption capacity for H_2_S and NH_3_ gases, and the amino functionalization also imparted a good adsorption capacity for acetaldehyde. In addition, the composite material was also functionalized simultaneously with Cu^2+^ and amino groups, and was proved to adsorb H_2_S, NH_3_, and acetaldehyde gases at the same time. The article by Onota et al. [334] presents a CaP for malodor adsorption that is derived from renewable sources. In this work, calcium carbonate from corbicula shell waste was converted into brushite by dissolution with phosphoric acid and reprecipitation with ammonia. Some of the products were proven to have a good adsorption capacity for the malodorous trimethylamine gas, although the mechanism of adsorption was not cleared. Finally, in the article by Rastrelli et al. [335], the deodorant effect of HA was tested in vivo. This work evaluated the sweat production from volunteers in controlled conditions treated with a test emulsion formulation containing magnesium- and zinc-doped HA in conjunction with zinc pidolate. It was found that the HA formulation led to a production of sweat that was ca. 30% less than the treatment with an HA-free placebo formulation, although the single contributions of HA and zinc pidolate were not discriminated. Therefore, the work implies that HA could also have an antiperspirant effect.

The number of patents about CaPs as deodorant materials is more limited in comparison to other cosmetic applications, as only seven patents were registered in the last two decades (Table 7). Interestingly, the majority of these patents were deposited between 2001 and 2011 [254,336,337,338,339], suggesting that the interest in this field is not rising. In agreement with the scientific literature, for this application, almost all the examined patents protect the use of CaPs as absorbing materials capable of catching volatile malodorous substances through non-covalent interactions, limiting their volatilization [254,337,339,340]. However, a few of them also claim an antiperspirant or antimicrobial action exerted by aluminum, copper, magnesium, iron, silver, or zinc oxide doping in HA [336,338,341].

It should be noted that for this application HA is the only CaP phase used, and its doping with foreign elements has great relevance. Indeed, as reported in scientific literature, aluminum is usually used in antiperspirant products, while other elements—often in the form of oxides—are used as bactericidal/deodorizing agents. For this reason, doped HAs reported in patents can have both odor-absorbing and bactericidal effects. Finally, even though the number of research works showing the potential of CaPs as a deodorant ingredient is high, the number of patents in the field is relatively low.

## 4. Conclusions and Perspectives

Cosmetic products are applied on the human body to clean, beautify, or alter the appearance without affecting body physiology or functions. Despite the recent concerns regarding the health and environmental hazards of cosmetics posed, for example, by heavy metal or paraben exposure, the cosmetics market is ever-growing. However, the rising awareness of these hazards is pushing towards the development of innovative, natural, sustainable, and safe cosmetics using non-toxic ingredients that have high biocompatibility and biodegradability. For this purpose, being one of the most used classes of biocompatible and biodegradable materials in medicine, CaPs represent an excellent alternative to several currently used ingredients. The main applications of CaPs in cosmetics that we have found searching through patents and scientific papers are as tooth whiteners, make-up products for skin cleaning and beautification, deodorants and agents for hair dyes. Among the different CaP phases, HA is the most studied due to its intrinsic similarity with the mineral phase of bone and tooth.

The scientific literature on this topic is still in its infancy, while, interestingly, the use of CaPs in cosmetics has gained considerable interest from the industry, leading to a massive production of patents to protect industrial research work and know-how. This discrepancy can be due to the fact that (i) cosmetic formulations are very complex and rich in coadjutants, thus making it difficult to study in a systematic way since the individual effect and influence of each ingredient cannot be perfectly assessed, and that (ii) there is a deficiency of well accepted analytical protocols to evaluate the efficacy of raw materials for cosmetic applications. In this view, the current lack of scientific literature suggests that there are still unexplored opportunities for material scientists to design new CaP-based materials as well as a new useful methodology for testing in this field.

Regarding the different applications of CaPs in cosmetics, some perspectives can be drawn. For tooth whitening, since all CaP products for remineralization also have a whitening effect, it is likely that the focus of this research will be on the development of more efficient remineralization materials than on bleaching ones. In the field of sunscreen, the use of ingredients able to improve the SPF of a product represents an interesting approach in the development of safer and sustainable sunscreens. The so-called “SPF boosters” are raw materials that, with different mechanisms of action, can enhance the SPF of a solar formulation, and thus allow for a reduction in the concentration of chemical and physical filters. Therefore, we can envisage more consideration for the use of CaPs as boosters and thus more studies on their efficacy with organic and inorganic UV filters. For skin care applications, the abrasive properties of CaPs can make them interesting products for skin cleaning, as well as sebum adsorbent materials, and their efficacy should be studied and optimized. Furthermore, nanometric CaPs can act as biocompatible stabilizers for Pickering emulsions and could substitute the currently used emulsifying agents. In application for hair care, CaPs can act as vehicles to deliver dyes, nutrients, and other useful molecules to the follicle and to hair skin, while, for deodorants, the research effort could be addressed by tuning the surface chemistry of CaPs to enhance their sorption properties and to fully substitute modern deodorants. In addition, the capability of CaP NPs to act as antiperspirants through pore blocking should be studied in more detail.

Some general trends can be also anticipated. Firstly, we envisage a growth in the use of CaPs from biogenic sources, and in particular from by-products, as this represents a way to prepare sustainable and natural cosmetics, as sustainability and naturality have already become two keywords of the cosmetics market trend worldwide. On this topic, several studies have proved that CaPs produced from natural sources have the same properties attributed to synthetic CaPs. Secondly, we predict that, similar to what occurred for CaPs in the medical field, the relevance of ion doping as a strategy to impart new properties will become more and more relevant. Indeed, the high number of works that employ this strategy show that doping could be a successful method to enhance the efficacy of CaPs for cosmetic application. Another opportunity that has been neglected until now is the use of alternative CaP crystal phases to HA. Indeed, almost all of the research works reported in this review have been focused on HA, as it is the most used material in medicine and cosmetics. Different CaP phases have different properties, such as solubility, morphology, and surface chemistry, that could make them attractive for specific applications. Finally, as in nanomedicine, CaP NPs are becoming appealing materials for drug delivery, and the same features could be used in cosmetics to deliver nutraceuticals, biologically relevant molecules, dyes, and many other substances.

## Figures and Tables

**Table 2:** List of patents about calcium phosphates used to aid the delivery of biomolecules to skin.

Ref	Title	Applicants	Publication Year	Description
[164]	Ubiquinone-containing composition	Sangi Co	2007	Base material for ubiquinone stabilization. CaP: HA
[165]	Compositions comprising polyesters of biological origin and biocompatible inorganic compounds, and uses thereof in the cosmetics field	Bio On SpA	2019	Base material for delivery of ingredients to the skin. CaP: HA composite with polymers
[166]	A pH hybrid nanoparticle comprising calcium phosphate a preparation method thereof and a smart delivery vehicle for loading and delivery of bioactive agents	University-Industry cooperation group of Kyung Hee University	2019	Product for skin. CaP: non-disclosed composite particle with CaP and polymers
[167]	Compound composed of aliphatic polycarbonate and inorganic compound and application of compound in related fields of cosmetics	Zhongkai University of Agriculture and Engineering	2020	Base material for delivery of ingredients to the skin. CaP: non-disclosed composite particle with HA and polycarbonate

**Table 3:** List of patents about calcium phosphates as sunscreens.

Ref.	Title	Applicants	Publication Year	Description
[223]	Coated powdery material and cosmetics containing the same	Pola Chem Ind INC	2006	Powder for UV absorption. CaP: HA-coating on a multilayer material
[224]	Sunscreen cosmetics	Fancl Corp	2009	Sunscreen for UV absorption. CaP: HA
[225]	Organic-inorganic composite powder, a preparation method thereof, and a use of the same	Woongjin Coway Co., Ltd.	2011	Powder for UV absorption. CaP: HA-coating on a hollow particle
[226]	Sunscreen product comprising hydroxyapatite as physical filter	Kalichem Italia SRL	2012	Product for solar radiation protection. CaP: HA
[227]	Sunscreen Compositions Comprising Uniform, Rigid, Spherical, Nano porous Calcium Phosphate Particles and Methods of Making and Using the Same	Laboratory Skin Care Inc.	2016	Product for solar radiation protection. CaP: not disclosed
[228]	UV-filters, method of producing the same and their use in compositions, in particular sunscreens	Universidade Catolica Portuguesa	2017	Product for UV absorption in sunscreen and textiles. CaP: iron-doped HA + iron oxide
[229]	Physical solar filter consisting of substituted hydroxyapatite in an organic matrix	Consiglio Nazionale Delle Ricerche	2017	Product for solar radiation protection. CaP: HA doped with titanium and iron
[230]	Hydroxyapatite-transition metal composite preparation method thereof and material for blocking ultraviolet rays and visible rays comprising the same	Industry-Academic Cooperation foundation Gyeongsang National University	2018	Product for UV and Vis shielding. CaP: iron-doped HA + iron oxide
[231]	Physical sunscreen comprising hydroxyapatite or modified hydroxyapatite obtained from fisheries and aquaculture waste, process for its production and photoprotective compositions comprising it	Consiglio Nazionale Delle Ricerche	2020	Product for solar radiation protection and photoprotective boost effect. CaP: HA derived from natural sources doped with metal ions, TCP + metal oxides
[232]	Calcium phosphate-folic acid composite particles, preparation method and application thereof	Hunan Yujia Cosmetics Manufacturing Co ltd	2020	Product for UV absorption and scattering. CaP: non-disclosed composite with folic acid

**Table 4:** List of patents about calcium phosphate as skin cleansers.

Ref	Title	Applicants	Publication Year	Description
[249]	Creamy apatite face cleanser	Kazushi Hirota, Katsunari Nishihara	2002	Skin cleaner cream. CaP: HA
[250]	Cosmetics	Sangi Co	2001	Product for skin renewal. CaP: amorphous HA
[251]	Skin care preparation	Noevir Co., Ltd.	2002	Product for skin renewing. CaP: HA
[252]	Skin care preparation	Fancl Corp	2003	Product for sebum control. CaP: HA
[253]	Sebum secretion control kit	Fancl Corp	2004	Product for sebum control. CaP: HA
[254]	Sebum adsorbing powder and use thereof	Miyoshi Kasei Inc.	2004	Powder for sebum adsorption and deodorant. CaP: zinc oxide-coated HA
[255]	Porous particle of synthetic resin bonded with hydroxy apatite particle, external preparation and cosmetics	Fancl Corp	2005	Product for sebum adsorption. CaP: HA coating on resin particles
[256]	Synthetic resin porous particle combined with hydroxyapatite particle, external preparation, and cosmetics	Fancl Corp	2005	Product for sebum adsorption. CaP: HA coating on resin particles
[257]	Application method of nano calcium phosphate like salt for cosmetics product	Sun Zhenlin	2006	Product for skin cleansing. CaP: TCP, HA, OCP, calcium pyrophosphates
[258]	Cosmetics	Sekisui Plastics Co., Ltd., Shiseido Co., Ltd.,	2007	Product for sebum adsorption. CaP: ACP coating of mica particles
[259]	Make-up composition	Procter & Gamble	2007	Product for sebum control. CaP: HA coating of zinc oxide
[260]	Cosmetics for cleaning skin	Mandom Corp	2008	Product for skin cleansing, sebum removal, and skin smoothing. CaP: surface-modified HA
[261]	Cosmetics for cleansing skin	Mandom Corp	2009	Product for skin cleansing, sebum removal, and skin smoothing. CaP: surface-modified HA
[262]	Skin-cleaning agent composition	Mandom Corp	2010	Product for skin cleansing. CaP: HA
[263]	Cosmetics and cleansing agent having detox function	Kankyo Hozen Kenkyusho: Kk,	2011	Product for skin cleansing, and deodorant. CaP: non-disclosed CaP coating on titanium dioxide
[264]	Gommage cosmetics material	Mikimoto Pharmaceut Co., Ltd.	2012	Gommage product with sebum control. CaP: HA
[265]	Biomass nerve soothing facial mask liquid	Gao Xinwen	2019	Liquid for facial mask. CaP: HA

**Table 6:** List of patents about calcium phosphates in hair care.

Ref	Title	Applicants	Publication Year	Description
[315]	Temporary hair dye cosmetics composition	Cosmax INC	2015	Solid temporary hair dye product. CaP: HA
[316]	Hair dye composition	Kuriya Yumi: Kk	2013	Temporary hair dye product. CaP: HA particles adsorbed on titanium dioxide powder

**Table 7:** List of patents about calcium phosphates as deodorants.

Ref	Title	Applicants	Publication Year	Description
[254]	Sebum adsorbing powder and use thereof	Miyoshi Kasei Inc.	2004	Powder for sebum and fatty acids adsorption and deodorization. CaP: zinc oxide-coated HA
[336]	Antimicrobial fine particle, method for producing the same and cosmetics or antimicrobial insecticide containing the antimicrobial fine particle	Kyowa Industrial Co., Ltd., Sangi Co., Ltd., Suzuki Yushi Kogyo Kk	2005	Deodorant, antimicrobial, antiperspirant, sebum absorbing product. CaP: HA
[337]	Non-aqueous powder aerosol	Nivea Kao KK	2005	Deodorizing powder, specifically for low MW fatty acids. CaP: HA
[338]	Bactericidal/deodorizing agent	Asahi Shokai: Kk	2007	Deodorant and sterilizing product. CaP: silver-HA composite
[339]	Body odor suppressing agent and cosmetics product compounded therewith	Miyoshi Kasei Inc.	2011	Deodorizing powder. CaP: HA
[340]	Bentonite deodorant	Tianjin Zhongtian Jingke Technology Co., Ltd.	2018	Deodorant product. CaP: HA
[341]	Antiperspirant and deodorant compositions	Kalichem S.r.l.	2020	Antiperspirant and deodorant product. CaP: HA doped with aluminum, zinc, magnesium, zirconium, titanium, copper, silver, or iron

## Data Availability

The data presented in this study are available on request from the corresponding author.
